# Extremal quantum correlation generation using a hybrid channel

**DOI:** 10.1038/s41598-023-43811-6

**Published:** 2023-10-03

**Authors:** Atta ur Rahman, Hazrat Ali, S. M. Zangi, Cong-Feng Qiao

**Affiliations:** 1 School of Physics, University of Chinese Academy of Science, Yuquan Road 19A, Beijing, 100049 China; 2 Abbottabad University of Science and Technology, Havellian, KP 22500 Pakistan; 3 School of Physics and Astronomy, Yunnan University, Kunming, 650500 China

**Keywords:** Quantum information, Quantum physics, Qubits

## Abstract

The preservation of quantum correlations requires optimal procedures and the proper design of the transmitting channels. In this regard, we address designing a hybrid channel comprising a single-mode cavity accompanied by a super-Gaussian beam and local dephasing parts based on the dynamics of quantum characteristics. We choose two-level atoms and various functions such as traced-distance discord, concurrence, and local-quantum uncertainty to analyze the effectiveness of the hybrid channel to preserve quantum correlations along with entropy suppression discussed using linear entropy. The joint configuration of the considered fields is found to not only preserve but also generate quantum correlations even in the presence of local dephasing. Most importantly, within certain limits, the proposed channel can be readily regulated to generate maximal quantum correlations and complete suppression of the disorder. Besides, compared to the individual parts, mixing the Fock state cavity, super-Gaussian beam, and local dephasing remains a resourceful choice for the prolonged quantum correlations’ preservation. Finally, we present an interrelationship between the considered two-qubit correlations’ functions, showing the deviation between each two correlations and of the considered state from maximal entanglement under the influence of the assumed hybrid channel.

## Introduction

Since the advent of quantum theory, quantum entanglement has captured the interest of physicists. Entangled quantum systems can exhibit peculiar behavior and properties that appear to contravene our sensible conceptions of how the universe functions^1^. A strange, paradoxical phenomenon known as quantum entanglement illustrates how two subatomic particles can be closely connected despite being billions of light-years apart^2^. Despite the great distance between them, a change brought about in one will impact the other. John Bell, a physicist, proposed in 1964 that even when the particles are very far away, such transformations can happen instantly^3,4^. In this regard, one of the pathways to studying this non-classicality is the dynamics of quantum systems, where various types of dynamical aspects such as classical/quantum semi-groups, positive maps, open and closed non-local systems are studied in Ref.^5^ Besides this, the dynamics of open quantum systems is needed to uncover various aspects and phenomenon. For instance, quantum Zeno effects leading to the characterization of mixedness of an electronic state^6^, the analysis of near-resonant states along with computing the reduced equilibrium state of open systems^7^, based on Krotov approach an efficient optimal-control is designed using the concept of non-Markovian dynamics, while the detailed scaling dynamical behaviors of quasi-particles with Markovian dynamical maps all are investigated using the open quantum system formalism^8^.

Furthermore, quantum correlations are considered a significant part of quantum information theory^9^. Entanglement is one of the most important types of correlation, though it is by no means the only relevant category. Several recent studies have shown that quantum correlations beyond entanglement may be capable of overcoming the corresponding classical constraints in a wide range of situations^9,10^. One such quantum correlation is local quantum uncertainty (LQU) introduced by Girolami et al., which can estimate pure quantum correlations, but not their classical equivalents^11^. This way of measuring is of the quantum discord form, but it has the benefit that the relative assessments do not require the time-consuming optimization process. The LQU measure only has a closed formula for $2 \otimes d$ quantum systems and was initially developed to compute bipartite quantum correlations^12^. Note that the LQU metric computes the degree of uncertainty raised in a provided non-local system because of its non-commutativity with corresponding local observables. This quantifier can be determined by taking the minimum of the skew information. Besides, instead of its significance as a reliable non-classicality measure, LQU can also be readily related to the concept of quantum Fisher information^13^, which makes it an easy-to-access key for quantum metrology protocols. In Ref.^14^, pairwise quantum correlation beyond entanglement in a spin-square complex system has been successfully revealed using LQU. In the system of a two-qubit Heisenberg spin model, various parameters and associated optimal tuning of anisotropy, strength, and frequency of the external magnetic field, and the system’s purity are determined for the preservation of quantum correlations using LQU^15^. In Refs.^16–18^, the impact of the external magnetic field, and local dephasing effects on various quantum systems, such as the Heisenberg spin model, quantum dot, and qubit–qutrit systems with the associated degree of quantum correlations are successfully revealed using LQU measure. Besides, quantum discord has been discovered to be another important quantum correlation, however, because of the hindrance in the numerical manipulation, its geometric quantum discord (GQD) form has been presented^19^. GQD can be defined as the minimum distance between the given and classical states using p-norms as trace-norm, employed to define trace distance discord (TDD)^20^ and the Hilbert–Schmidt norm^21^. Recently, GQD has been affected by the local reversible operations on its subsystem^22^, hence, TDD may be illustrated as a consistent quantum correlation measure.

In quantum information processing, nearly all the quantum phenomena and related efficiency are based on the degree of non-classical correlations between the sub-systems of a composite state^23^. For instance, quantum mechanical protocols such as quantum imaging^24^, teleportation^25^, metrology^26^, remote state preparation^27^, key-distribution^28^, cryptography^29^, and quantum state tomography^30,31^ are few to name. In this regard, various schemes for quantum correlation generation have been proposed theoretically and experimentally. For example, for pairwise entangled electron generation, a surface polariton-supporting medium has been proposed^32^. In another case, a common phonon reservoir has been utilized to generate non-classical correlations between two excitonic quantum dot qubits^33^. The time evolution of the generated two-qubit entanglement and discord from an uncorrelated system when exposed to a common thermal or squeezed reservoir is investigated in Ref.^34^. An experimentally applicable scheme for the generation of a multi-spatial-mode quantum light source using a non-degenerated four-wave mixing procedure in a thermally controlled atomic vapor cell has been demonstrated in Ref.^35^. Besides, another practical scheme, the generation of non-classical correlations and practically controllable parameters such as photon detuning and power of the pumping source, is presented^36^. Other examples, where the generation of quantum correlations has been discussed can be seen in Refs.^37–39^. In this regard, we implement a Fock-state cavity when driven by a super-Gaussian field for the generation of quantum correlations in a two-qubit state.

Because the features of cavities fitted with Gaussian reflectivity mirrors are well-known, these reflectors have received the most attention^40^. The utilization of super-Gaussian reflectivity mirrors has recently been demonstrated to provide a superior filling of the active material and produce modes with higher energy^40^. Recently, the super-Gaussian field has been used to study the classical transport theory and the generation of thermal instability in laser-plasma materials^41^. The super-Gaussian field has also been found to be resourceful in demonstrating high-field THz radiations using quasi-static fluid theory^42^. A detailed illustration of specular reflection and for understanding the velocity filtering and deceleration phenomena has been done for the neutral ground-state molecules when exposed to a super-Gaussian optical beam^43^. The authors of Ref.^44^ have studied an entropic formalism for understanding the free dynamics of Gaussian beams when subjected to lenses, while also considering its extension to super-Gaussian beams. Besides, atomic microscopy regulated by the absorption spectrum of a weak probing field composed of two reinforced super-Gaussian beams is found to be significant in advanced high-tech and nano-lithographic applications^45^. It is now evident that the super-Gaussian fields have been studied significantly in various fields, however, they have not yet been investigated in the case of the dynamics of open quantum systems. Therefore, its relevance to the preservation of quantum correlations might be of great importance in quantum systems, which we will discuss in this work.

The experimental realization of the Fock state cavity scheme when interacting with excited atoms has been presented in Ref.^46^. A model comprising coupling between three-level atoms, quantized cavity mode, and a classical field is demonstrated in Ref.^47^ which proposed the generation of large Fock states in the cavity mode, the reconstruction of the Wigner function of an arbitrary cavity field state, and entanglement generation. In Ref.^48^, a one-photon state is shown to exhibit negligible purity losses in the cavity, however, the purity states of the system were highly degraded by the degree of damping in the cavity. Motivated by this, we propose implementing a Fock state cavity scheme for the dynamics of a two-qubit state and investigating its impact on the generation of quantum correlations.

In this work, we aim to explore the advantages of the simultaneous application of the Fock state cavity, super-Gaussian beam, and local dephasing while considering the case of a two-qubit system. The Fock-state cavity is observed to induce quantum correlations, however, the performance of the Fock-state cavity field may be enhanced when superimposed with a super-Gaussian field. As any realistic quantum information processing protocol without decoherence effects is assumed to be ideal, we include two independent classical dephasing fields. This would allow us to examine how much our considered model can bear with external decoherence effects and remain efficient for quantum mechanical protocol deployment. Therefore, in the current work, we jointly apply the Fock-state cavity, the super-Gaussian field, and two independent classical channels. Besides entanglement measurement using concurrence (CN), we also investigate the presence and strength of other quantum correlations beyond entanglement, such as LQU and TDD. In such quantum setups, entropy has a prominent place and cannot be disregarded in any quantum information processing protocols. Hence, to understand the impact of joint Fock-state cavities and super-Gaussian fields on the behavioral dynamics of the two-qubit state, we also utilize linear entropy (LE). In addition, the current quantum setup will be characterized by various related parameters. Therefore, the impact of each parameter will be characterized independently and its role in quantum correlations’ generation and entropy suppression will be studied in depth. Finally, we will propose the best suiting scheme, fields, and parameters characterization for the higher degree of two-qubit non-classical correlations generation.

This work is presented as: In “Introduction”, we present the physical model of the assumed system and cavity along with an introduction to the measurement of quantum correlations and entropy. “Results” gives a detailed analysis of the results obtained and in “Conclusion and outlook”, we summarize this work.

## Physical model

### Two-level atom system and Fock state cavity

We consider imposing a single-mode cavity field resonantly on two two-level atoms, initially prepared in the Fock state mode. Using the rotating-wave approximation approach, the relative Hamiltonian of the current configuration can be written as^37^:

1 $$\begin{aligned} {\mathscr {H}}=\sum _{\Omega =1}^{2}\mathscr {G} [a S_{+}^{(j)}+a^{\dagger }S_{-}^{(j)}]+2k [S_{-}^{(1)}S_{+}^{(2)}+S_{+}^{(1)}S_{-}^{(2)}]+\Omega [ S_{z}^{(1)}S_{z}^{(2)}], \end{aligned}$$

**Figure 1:**
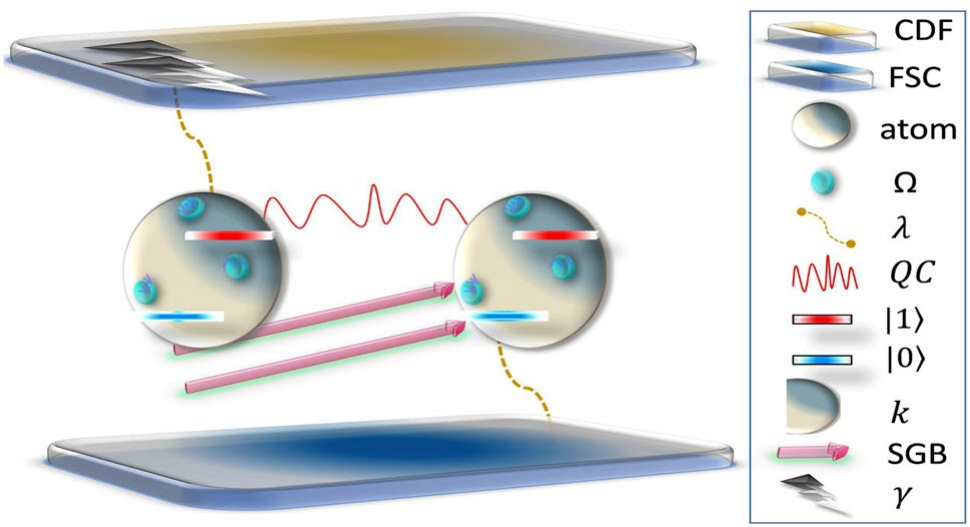
Shows the schematic diagram of the two two-level atoms exposed to Fock state cavity (FSC), classical dephasing field (CDF), and super-Gaussian beam (SGB). Note that $\Omega$ is the Ising interaction, $\lambda$ is the coupling strength between the hybrid channel and atoms, QC denotes the quantum correlations between the two atoms, $|0\rangle (|1\rangle )$ are the ground(excited) states, *k* is the dipole–dipole interaction while $\gamma$ is the photon state.

where $\mathscr {G}$ is the coupling strength intensity, *a*($a^{\dagger }$) is the creation(annihilation) operator acting on single-mode cavity field, $S _{\pm }=\left( S _{x}\pm S _{y}\right) /2$ (with $\{ S _{x},S _{y},S _{z}\}$ are the Pauli matrices), *k* and $\Omega$ regulates the dipole–dipole and Ising coupling strengths, correspondingly. Besides, operator $I=a^{\dagger }a+\frac{1}{2}\left( S_{z}^{(1)}+S _{z}^{(2)}\right)$ is a constant of motion and can be assumed as the number of excitations brought up in the system. Therefore, in the system’s subspace $I\equiv \gamma +1$, the chosen basis are $\Bigl \{ \bigl |\gamma +1,00\bigr \rangle ,\bigl |\gamma ,01\bigr \rangle ,\bigl |\gamma ,10\bigr \rangle ,\bigl | \gamma -1,11\bigr \rangle \Bigr \}$ where $\bigl |\gamma \bigr \rangle$ describes the state of $\gamma$ photons, as shown in Fig. 1. In addition, $\bigl |0\bigr \rangle$ and $\bigl |1\bigr \rangle$ represent the ground and excited state of two two-level atoms.

Next, the eigenvalues of Eq. (1) can be written as:

$$\begin{aligned} \mathscr {L}_1=&\Omega ,&\mathscr {L}_{2}=&-(2k+\Omega ),&\mathscr {L}_{3,4}=&k\pm \beta ,&\beta =&\sqrt{\mathscr {G}^{2}[2+4 \gamma ]+\left[ \Omega -k\right] ^{2}}. \end{aligned}$$

Besides, the eigenvectors of the Hamiltonian (Eq. 1) obtained has the form:

$$\begin{aligned} \left| \nu _{1}\right\rangle =&\frac{1}{a_1}\left( -\sqrt{\gamma } \bigl |\gamma +1,00\bigr \rangle +a_2\bigl |\gamma -1,11\bigr \rangle \right) ,&\left| \nu _{2}\right\rangle =&\frac{1}{\sqrt{2}}\left( -\bigl |\gamma ,01 \bigr \rangle +\bigl |\gamma ,10\bigr \rangle \right) ,\\ \left| \nu _{3}\right\rangle =&\frac{1}{2a_3} \left( 2\mathscr {G}a_2\bigl |\gamma +1,00\bigr \rangle +2\mathscr {G}\sqrt{\gamma }\bigl |\gamma -1,11 \bigr \rangle \right. +\left. (a_4\left[ \bigl |\gamma ,01\bigr \rangle +\bigl |\gamma ,10\bigr \rangle \right] \right) , \\ \left| \nu _{4}\right\rangle =&\frac{1}{2a_5} \left( 2\mathscr {G}a_2\bigl |\gamma +1,00\bigr \rangle +2\mathscr {G}\sqrt{\gamma }\bigl |\gamma -1,11 \bigr \rangle \right. -\left. a_6\left[ \bigl |\gamma ,01\bigr \rangle +\bigl |\gamma ,10\bigr \rangle \right] \right) , \end{aligned}$$

where

$$\begin{aligned} a_1&=\sqrt{1+2\gamma },&a_2&=\sqrt{\gamma +1},&a_3&=\sqrt{\beta a_4},&\\ a_4&=\beta +(k-\Omega )),&a_5&=\sqrt{\beta (\beta -(k-\Omega ))},&a_6&=\beta -(k-\Omega ).&\end{aligned}$$

The thermal state density matrix at equilibrium temperature *T* can be obtained by $\rho _{AB} (T)=1/M\exp \left( -\mathscr {H} / \mathscr {U} T \right)$ with the partition function $M={\textrm{Tr}}\left[ \exp \left( -\mathscr {H} / \mathscr {U} T \right) \right]$ and $\mathscr {U}$ denotes the Boltzmann’s constant ($\mathscr {U} =1$). Finally, the reduced density matrix of the system is obtained by taking the trace over the field and the associated matrix elements as

2 $$\begin{aligned} \rho _{AB} (0, T)=\left( \begin{array}{cccc} \rho _{11} &{} 0 &{} 0 &{} 0 \\ 0 &{} \rho _{22} &{} \rho _{23} &{} 0 \\ 0 &{} \rho _{32}^* &{} \rho _{33} &{} 0 \\ 0 &{} 0 &{} 0 &{} \rho _{44} \end{array} \right) , \end{aligned}$$

where

3 $$\begin{aligned} \rho _{11}= & {} \frac{1}{M}\left( \mathscr {X}_1\exp {[}\mathscr {W}_1]+\mathscr {X}_2\exp [\mathscr {W}_2]\left[ \cosh \left( \mathscr {W}_3\right) +\mathscr {X}_3\sinh \left( \mathscr {W}_3\right) \right] \right) , \nonumber \\ \rho _{44}= & {} \frac{1}{M}\left( \mathscr {X}_2\exp {[}\mathscr {W}_1]+\mathscr {X}_1\exp [\mathscr {W}_2]\left[ \cosh \left( \mathscr {W}_3\right) +\mathscr {X}_3\sinh \left( \mathscr {W}_3\right) \right] \right) ,\nonumber \\ \rho _{22}= & {} \rho _{33}=\frac{1}{2M}\left( \exp [\mathscr {W}_4]+ \exp [\mathscr {W}_2]\left[ \cosh \left( \mathscr {W}_3\right) -\mathscr {X}_3\sinh \left( \mathscr {W}_3\right) \right] \right) , \nonumber \\ \rho _{23}= & {} \rho _{32}^{*}=-\frac{1}{2M}\left( \exp {[}\mathscr {W}_4]+ \exp [\mathscr {W}_2]\left[ \cosh \left( \mathscr {W}_3\right) -\mathscr {X}_3\sinh \left( \mathscr {W}_3\right) \right] \right) , \nonumber \\ M= & {} \exp [\mathscr {W}_1]+2\exp [\mathscr {W}_2]\cosh \left( \mathscr {W}_3\right) +\exp [W_4] . \end{aligned}$$

Here

$$\begin{aligned} \mathscr {W}_1&=-\Omega /T,&\mathscr {W}_2&=-k/T,&\mathscr {W}_3&=\beta /T,&\mathscr {W}_4&=(\Omega +2k)/T,\\ \mathscr {X}_1&=\gamma /(1+2\gamma ),&\mathscr {X}_2&=1+\gamma /(1+2\gamma ),&\mathscr {X}_3&=(k-\Omega )\beta . \end{aligned}$$

### The super-Gaussian field

We assume the two-qubit state coupled with the Fock-state cavity is influenced by the super-Gaussian field. In quantum optics, a beam associated with a controlled field has the form $H(t) \propto \mathscr {G}(t) \exp [i \phi ]$ with $\mathscr {G}(t)$ is a function defining the coupling intensity of the field and influencing the shape of the beam ($\mathscr {G}(t)$ is also defined in Eq. (1) as the coupling strength) and $\phi$ denotes the phase of the fast laser pulse^49^. In this case, we consider a temporal super-Gaussian beam waveform that alternates between a smooth and pure flat-top shape. The dynamics of the super-Gaussian field distribution are determined by the associated super-Gaussian function, which defines the entire range of field distributions with sharp edges ranging from purely Gaussian to uniform flat-top. The control field, we are interested in, has the form^49^

4 $$\begin{aligned} \mathscr {G}(t)=\lambda \exp [-(t/\sigma )^p]. \end{aligned}$$

where $\lambda$ denotes the intensity of the coupling strength, the order of the super-Gaussian field is *p*, and $\sigma$ is the pulse rise time of the Gaussian beam spectrum.

Furthermore, the time-bandwidth product is computed utilizing temporal and spectral full widths at half maximum (FWHM) intensity widths. The basic form is dependent on time and spatial coordinates, and the angular frequency of the pulse and the wave number can be traced by taking the first derivative^50^. Finally, it is important to note that a variety of techniques can be used in experiments to shape pulses and process desired optical waveforms^51,52^. For example, the spatial light modulating programs^53^, micro-lithographic pattern technique^54^, the deformable mirrors^55^, and the acoustic–optic modulators^56^ are a few of them.

### Employment of two independent classical fields

Furthermore, no realistic quantum setup is free of external decoherence effects, therefore, we include a classical field driven by some disorder, resulting in noise. Let us consider a stochastic Hamiltonian governing the dynamics of a two-qubit system in two individual classical environments is written as^57^

5 $$\begin{aligned} \mathbb {H}(t)=\mathbb {H}_a(t) \otimes \mathbbm {1}_b+\mathbbm {1}_a\otimes \mathbb {H}_b(t), \end{aligned}$$

with the indicidual Hamiltonian has the form $\mathbb {H}_n(t)=\mathbbm {1}_n \mathscr {E}+\sigma ^z_n \delta \mathbb {O}_n(t)$ where $n \in \{a,b\}$, $\mathscr {E}$ denotes equal energy splitting, $\mathbbm {1}_n$ alongwith $\sigma ^z_n$ are identity and Pauli matrices. While $\delta$ is the coupling strength between the qubits and classical environments. The term $\mathscr {O}(t)$ remains the stochastic parameter of the classical fields regulating the flipping rate between $\pm 1$. The time-evolution of the system in the considered fields can be done by employing the time unitary operator as^58^

6 $$\begin{aligned} \mathbb {U}(t)=\exp \left[ -i \int _{t_0} ^{t} \mathbb {H}(f)df\right] . \end{aligned}$$

Finally, the time evolved state for the thermal density matrix $\rho (0, T)$ (2) can be obtained using the expressing

7 $$\begin{aligned} \rho (t, T)=U_a(t)U_b(t)\rho (0,T) U_a^{\dagger }(t)U_b^{\dagger }(t). \end{aligned}$$

The explicit form of the above expression has the form:

8 $$\begin{aligned} \rho (t, T)=\left( \begin{array}{cccc} \rho _{11} &{} 0 &{} 0 &{} 0 \\ 0 &{} \rho _{22} &{} \rho _{23} e^{2 i \delta t( \mathscr {O}_a-\mathscr {O}_b)} &{} 0 \\ 0 &{} \rho _{32}^* e^{-2 i \delta t (\mathscr {O}_a-\mathscr {O}_b)} &{} \rho _{33} &{} 0 \\ 0 &{} 0 &{} 0 &{} \rho _{44} \\ \end{array} \right) . \end{aligned}$$

### Classical noisy procedure

In order to account for noise, we assume that the classical fields are impacted by the Ornstein Uhlenbeck (OU) process, a stochastic procedure with ramifications in both finance and natural sciences. This physics term refers to the Brownian motion of the particles influenced by friction. In many quantum mechanical protocols, the OU approach, which is a static Gaussian–Markov operation causing OU noise, has been identified as one of several substantial causes of information losses^58^. Additionally, by subordinating a Gaussian white noise, the OU procedure was applied in order to examine linear stochastic dynamics under the involvement of Lévy white noise^59^. The authors of Ref.^60^ have identified an association between coherent quantum feedback OU processes and phase noise.

In the present case, OU noise is imposed on the dynamical map of the density matrix $\rho (0, T)$ given in Eq. (7). For the OU noisy consequences, we take a Gaussian process with zero-mean to explore the classical field *F*(*t*). Let the OU Gaussian process as second-order statistical operation with mean $\mathbb {X}$ along with the autocorrelation function *C* written as^58^

9 $$\begin{aligned} \mathbb {X}(t)=\mathbb {E}(\beta (t))=0, \,\text {and}\,\, C(t, {t}^\prime )=\mathbb {E}(\beta (t)\beta (t^{\prime })), \end{aligned}$$

where $\mathbb {E}({\textbf {.}})$ shows the average of all the realizations of $\beta (t)$ function. Furthermore, Gaussian processes in terms of its characteristic function can be written as

10 $$\begin{aligned} \mathbb {E}\left[ e^{\left( i\int _0^t J(s) \beta (s) ds \right) } \right] =\exp {\left( {-\frac{1}{2}\int _0^t\int _0^t J(s) C(s, s^{\prime }) J(s)^{\prime }ds ds^{\prime }} \right) }, \end{aligned}$$

where *J*(*t*) is a function of time. Keeping $J=n$ constant with time, Eq. (10) is yield as

11 $$\begin{aligned} \mathbb {E}\left[ \exp \left( \pm i \int _0^t \mathscr {B}(s)ds \right) \right] =\exp \left( -\frac{1}{2}n^2 \eta (t)\right) \,\text {with}\,\eta (t)=\int _0^t \int _{t_0}^t C(s-s^{\prime })ds ds^\prime . \end{aligned}$$

where $\eta (t)$-function is utilized to impose the local noise field onto the environments where $C(g, t-t^{\prime })=\frac{g}{2}\exp {\left( -g|t-t^{\prime }|\right) },$ and $g= 1/\tau$ with $\tau$ is the memory element and the correlation time, respectively. For simplicity, we take $\tau =t$.

Now, the $\eta$-function is obtained by inserting $C(g, t-t^{\prime })$ into Eq. (11) and we get

12 $$\begin{aligned} \eta (t)=\frac{1}{g}[gt  -1+\exp {[-gt ]}]. \end{aligned}$$

Finally, the ultimate density matrix for the two qubits influenced simultaneously by Fock state cavity, super-Gaussian and classical dephasing field is obtained as

13 $$\begin{aligned} \rho (f, T)=\Big \langle \Big \langle \rho (t, T) \Big \rangle _{\Phi _a(t)} \Big \rangle _{\Phi _b(t)}=\left( \begin{array}{cccc} \rho _{11} &{} 0 &{} 0 &{} 0 \\ 0 &{} \rho _{22} &{} \hat{\rho _{23}} &{} 0 \\ 0 &{} \hat{\rho _{32}}^* &{} \rho _{33} &{} 0 \\ 0 &{} 0 &{} 0 &{} \rho _{44} \\ \end{array} \right) , \end{aligned}$$

where $\Phi _n(t)=-1/2m^2\eta (t)$ with *m* being an integer and in the current case $m=\pm 2$ while $n=\{a,b\}$. Note that $\hat{\rho _{32}^*}=\hat{\rho _{23}}=\rho _{23} \exp [{-\frac{4 \left( g t+e^{g (-t)}-1\right) }{g}}].$

### Correlation measures

#### Trace measure of geometric discord

Paula used the trace norm (also known as the 1-norm) as a trustworthy geometric notation of quantum discord^61^. Analysis has been done to extract the expressions of trace distance quantum discord (TDD) for any arbitrary two-qubit *X* states and Bell-diagonal states^61,62^. The TDD for a two-qubit state $\rho _{AB}$ can be defined by

14 $$\begin{aligned} {\textrm{TDD}}= \frac{1}{2} \min _{\eta \in \varphi }||\rho _{AB}-{\varrho _{\textrm{AB}}}||_1, \end{aligned}$$

where $||\rho _{AB}-{\varrho _{\textrm{AB}}}||_1={\varrho _{\textrm{AB}}}\sqrt{(\rho _{AB}-{\varrho _{\textrm{AB}}})^\dag (\rho _{AB}-{\varrho _{\textrm{AB}}})}$ defines the trace distance. The distance between any given state that is a part of the set $\varphi$ of classical-quantum states and the quantum state $\rho _{AB}$ is estimated by TDD. A generic classical-quantum state ${\varrho _{\textrm{AB}}}\in \varphi$ may of the form ${\varrho _{\textrm{AB}}} = \sum _m p_m \,\Pi _{m,A}\otimes \rho _{m,B}$ with $\{m_k\}$ is the probability distribution, and $\Pi _{m,A}$ are the orthogonal projector corresponding to the qubit *A* and $\rho _{k,B}$ is the density matrix relative to the qubit *B*. The terms $\dfrac{\rho _{14}}{|\rho _{14}|}= e^{i\theta _{14}},\,\dfrac{\rho _{23}}{|\rho _{23}|}= e^{i\theta _{23}}$ i.e., the off-diagonal matrix elements can be eliminated through the local unitary transformations as

15 $$\begin{aligned} \left| 0 \right\rangle _A&\rightarrow \exp \left( \frac{ - i}{2}\left( \varpi _{14} + \varpi _{23}\right) \right) \left| 0 \right\rangle _A \,\,\text {and}\,\left| 0 \right\rangle _B\rightarrow \exp \left( \frac{ - i}{2}\left( \varpi _{14} - \varpi _{23}\right) \right) \left| 0 \right\rangle _B . \end{aligned}$$

The density matrix’s off-diagonal components also turn positive, and one gets

16 $$\begin{aligned} \rho _{AB} \rightarrow \tilde{\rho }_{AB} = \left( {\begin{array}{*{20}{c}} {{\rho _{11}}}&{}0&{}0&{}{\left| {{\rho _{14}}} \right| }\\ 0&{}{{\rho _{22}}}&{}{\left| {{\rho _{23}}} \right| }&{}0\\ 0&{}{\left| {{\rho _{23}}} \right| }&{}{{\rho _{33}}}&{}0\\ {\left| {{\rho _{14}}} \right| }&{}0&{}0&{}{{\rho _{44}}}\\ \end{array}} \right) . \end{aligned}$$

The above matrix in the Fano-Bloch representation can be rewritten as $\tilde{\rho }_{AB} = \sum \limits _{\alpha \beta } {Q_{\alpha \beta }} {\sigma _\alpha } \otimes {\sigma _\beta }$. The non-vanishing matrix elements $Q_{\alpha \beta }$ can be obtained by $$\begin{gathered} Q_{{11}} = 2(|\rho _{{23}} | + |\rho _{{14}} |),{\mkern 1mu} Q_{{22}} = 2(|\rho _{{23}} | - |\rho _{{14}} |),{\mkern 1mu} Q_{{33}} = 1 - 2(\rho _{{22}} + \rho _{{33}} ), \hfill \\ Q_{{03}} = 2(\rho _{{11}} + \rho _{{33}} ) - 1,{\mkern 1mu} Q_{{30}} = 2(\rho _{{11}} + \rho _{{22}} ) - 1. \hfill \\ \end{gathered}$$

Under local transformations, the TDD is invariant, and we get ${\textrm{TDD}}\left( \rho _{AB} \right) = {\textrm{TDD}}\left( {\tilde{\rho }_{AB} } \right) .$ The minimization in Eq. (14) has been worked out for a generic two-qubit–qubit *X* state^62^. It has been determined how to minimize the Eq. (14) for a two-qubit *X* state, and the TDD for the state $\rho _{AB}$ has the following form.

17 $$\begin{aligned} {\textrm{TDD}}=\frac{1}{2}\sqrt{\frac{Q_{11}^2 Q_{\textrm{max}}^2-Q_{22}^2Q_{\textrm{min}}^2}{Q_{\textrm{max}}^2-Q_{\textrm{min}}^2+Q_{11}^2-Q_{22}^2}}, \end{aligned}$$

where $Q_{\min}^2=\min \{Q_{11}^2,Q_{33}^2\},\,\text{with}\,Q_{\max}^2={\max } (Q_{33}^2, Q_{22}^2+Q_{30}^2)$ along with $(Q_{11}=2 (|\rho _{23}|),\,Q_{22}=2 (| \rho _{23}|),\, Q_{33}=-2 (\rho _{22}+\rho _{33})$, and $Q_{30}=2 (\rho _{11}+\rho _{22})-1).$ Note that in our case, the final density matrix whose entries are given in Eq. (13) has $\rho _{14}=\rho _{41}=0$.

#### Concurrence

Decoherence and disentanglement result from a quantum system’s interaction with its surroundings, which is an inevitable phenomenon. The level of entanglement in the state can be estimated using a measurement like concurrence. In the case of a density matrix state $\rho _{AB},$ defined by^37^, we opt for Wootter’s concurrence, given as

18 $$\begin{aligned} {\text {CN}}=\max \left( 0,\sqrt{\mu _4}-\sqrt{\mu _{3}}-\sqrt{\mu _{2}}-\sqrt{\mu _{1}}\right) , \end{aligned}$$

where $\mu _{i}$ denotes the eigenvalues of the Hermitian matrix $\varrho =\sqrt{\sqrt{\rho }\,\hat{\rho }\,\sqrt{\rho }}$ in decreasing order and $\hat{\rho }=\hat{\rho }_{AB}(S_{y}\otimes S_{y})\hat{\rho }^{*}(S_{y}\otimes S_{y})$ with $\hat{\rho }^{*}$ is the complex conjugate of the density matrix state $\hat{\rho }$.

The final density matrix is given in Eq. (13), and the mathematical results obtained using Eq. (18) for this matrix take the form

19 $$\begin{aligned} {\text {CN}}=\max [0, 2(\sqrt{\rho _{23} \rho _{32}}-\sqrt{\rho _{11} \rho _{44}})]. \end{aligned}$$

#### Local quantum uncertainty

Pure quantum correlations are captured by the LQU measure, but not their classical equivalents. The advantage of this quantum discord criterion is that measurements do not need to go through the laborious optimization process^63^. To proceed with the LQU measure, let us assume a two-qubit density matrix $\rho _{AB}$ with $O_A$ being a local Hermitian operator acting on the sub-system *A*, however, with a non-degenerate spectrum. The following is the description of the LQU metrics for subsystem *A*, optimizing global local observables acting on *A*, with unique eigenvalues^64^

20 $$\begin{aligned} {\text {LQU}} \equiv \min _{O_A} \mathscr {I}(\rho _{AB}, O_A \otimes I_2)\ . \end{aligned}$$

Here, $\mathscr {I}(\rho _{AB}, O_A \otimes I_2)=-\frac{1}{2}\textrm{Tr}([\sqrt{\rho _{AB}}, O_A \otimes I_2]^{2})$ is the Wigner–Yanase Skew Information (WYSI)^65,66^, with $I_2$ is the $2 \times 2$ identity matrix acting on sub-system *B*^67^. The skew information plays a significant role in quantum information theory and is essential for many application domains. This idea was developed to quantify the uncertainty in mixed states^65^. Fisher information, a statistical concept that is fundamental to both statistical estimation theory and quantum metrology, is another statistical concept that underlies skew information^66^. The analytical estimation of LQU is performed using a minimization protocol over the set of all observables acting on subsystem *A* of the composite system *AB*. For qubit–qubit systems, the minimization of LQU in Eq. (20) results in a closed formula having the form

21 $$\begin{aligned} {\text {LQU}} = 1 - \max \left( {{\Theta _{1}},{\Theta _{2}},{\Theta _{3}}} \right) , \end{aligned}$$

where $\Theta _{i=1,2,3}$ are the obtained eigenvalues from the $3\times 3$ symmetric matrix $\mathscr {U}_{ij}$ having the elements

22 $$\begin{aligned} \Big (\mathscr {U}\Big )_{ij} \equiv \textrm{Tr} \left[ \sqrt{\rho _{AB}}\Big (S_{A,i}\otimes {I}_{B}\Big )\sqrt{\rho _{AB}}\Big (S_{A, j}\otimes {I}_{B}\Big )\right] , \end{aligned}$$

with $S^A _{i} (i=x,y,z)$ are the spin Pauli matrices.

#### Linear entropy

The mixedness of quantum states is assessed using linear entropy, which is a straightforward scalar field to evaluate. One can compute the von Neumann entropy by $S(\rho _{AB})=-\hbox {Tr}[\rho _{AB}\log _{2}\rho _{AB}]$^68^. Next, approximating the $\log _{2}\rho _{AB}$ of $S(\rho _{AB})$ with the first-order term i.e., $\rho _{AB}-1$ in the Mercator series given in Ref.^69^, one may get

23 $$\begin{aligned} {\text {LE}}=-{\text {Tr}}[\rho _{AB}\log _2\rho _{AB}] \rightarrow -{\text {Tr}}[\rho _{AB}(\rho _{AB}-1)] ={\text {Tr}}[\rho _{AB}-\rho _{AB}^2]=1-{\text {Tr}}[\rho _{AB}^2]. \end{aligned}$$

For the next-to-last equality, the density matrix’s unit trace feature (${\text {Tr}}[\rho _{AB}]=1$) is used.

## Results

In this section, we give the details of the obtained results for the generation and dynamics of quantum discord, non-classical correlations, entanglement, and entropy using TDD, CN, LQU, and LE measures given in Eqs. (17), (18), (21) and (23). The results are based on the final density matrix’s elements given in Eq. (13) when the Fock state cavity is driven by a super-Gaussian field already illustrated in Eq. (4). Please see the physical model for the current configuration shown in Fig. 1. In the following, we characterize the TDD, LQU, CN, and LE functions by different parameters and observe the resultant generation and dynamics of two-qubit quantum correlations and entropy, respectively.

### The impact of photon state of the Fock-state cavity

**Figure 2:**
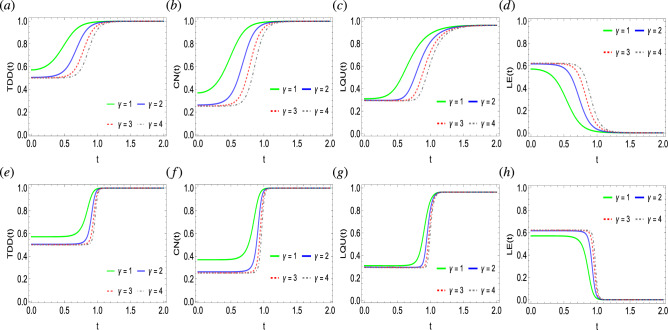
Dynamics of trace-distance discord (**a**,**e**), concurrence (**b**,**f**), local quantum uncertainty (**c**,**g**), and linear entropy (**d**,**h**) as functions of photon state $\gamma$ against time in a system of two qubits simultaneously influenced by Fock-state cavity, classical dephasing and super-Gaussian field when the order of the super-Gaussian field is set to $p=2$ (upper panel) and $p=8$ (lower panel). Other parameter settings are kept as: $k/\Omega /\sigma =1$, $T=0.5$, $\lambda =2$, $g=10^{-4}$.

Figure 2 explores the impact of the Fock-state cavity, super-Gaussian, and two independent classical noisy fields on the generation and time evolution of quantum correlations and entropy, when the TDD, CN, LQU, and LE functions are primarily characterized by photon state $\gamma$. It can be readily seen that initially, the state has non-zero discord, non-classical correlations, and entanglement, as {TDD, CN, LQU}$\ne 0$. However, it is also noticeable that the state is not free of entropy, as seen in $\text {LE}>0$. With time, the two-qubit quantum correlations get generated but at different rates. For example, the TDD function remains more enhanced and becomes easily developed. In comparison, the entanglement CN function follows TDD and seems to generate with time faster. Compared to the TDD and CN functions, the LQU measure encounters lesser non-classical correlations. Hence, suggesting the strengthened nature of the trace geometric quantum discord TDD compared to the entanglement and non-classical correlations functions CN and LQU. Besides, the LE function behaves opposite to the quantum correlations functions and continuously gets suppressed with time, hence, suggesting an inverse correlation between them. It is also noticeable that for the given $\gamma$ values, the slopes of the TDD and LQU functions seem more converged than those of the CN function. This suggests that the photon state $\gamma$ highly impacts the CN metric when compared to the TDD and LQU functions. Besides, the increasing photon strength seems to inversely affect the generation of quantum correlation and suppression of the entropy. For example, for $\gamma =\{2, 3, 4\}$, the quantum correlations generation takes place from lower initial levels, and the initial entropy in the state is at the highest. However, the opposite can be seen when the photon state parameter $\gamma =1$ i.e. kept minimum where quantum correlations functions approach their final saturation levels faster and start from higher initial levels. Moreover, for the photon state values, each quantum correlation function and entropy measure achieves a single saturation level, therefore, showing in-dependency between them. The order of the super-Gaussian field has also been found to influence the behavioral generation of quantum correlations. For the case of decreased order $p=2$, the generation of quantum correlations and the associated suppression of the entropy increases monotonically. For the higher order of the super-Gaussian field $p=8$, the TDD, CN, LQU, and LE functions evolve at a constant rate, however, at a certain point, a sudden transition of generation of quantum correlations to higher values and suppression of the entropy occurs. In addition, the initial, as well as final saturation levels of the LQU function, remain lower than the relative TDD and CN functions’ levels. Hence, suggesting the enhanced nature of quantum discord and entanglement functions compared to the LQU. The impact of photon state on the behavioral dynamics of quantum correlations between two nitrogen-vacancy centers, when subjected to photonic crystal cavities, has been investigated in Ref.^70^, however, the initial quantum correlations in the state were partially lost with time. In comparison, our physical model when primarily defined by the photon state avoids quantum correlations decay but generates it in the state.

### The impact of coupling strength of the super-Gaussian beam

**Figure 3:**
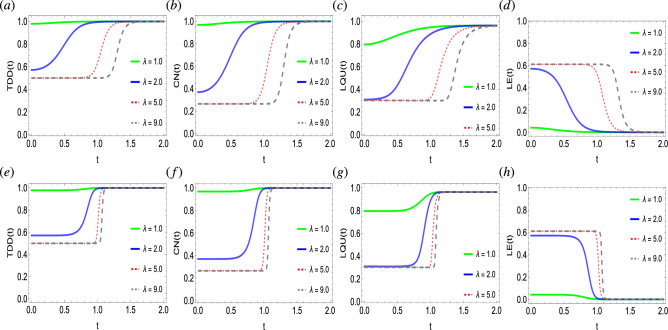
Dynamics of trace-distance discord (**a**,**e**), concurrence (**b**,**f**), local quantum uncertainty (**c**,**g**), and linear entropy (**d**,**h**) as functions of coupling strength $\lambda$ against time in a system of two qubits simultaneously influenced by Fock-state cavity, classical dephasing and super-Gaussian field when the order of the super-Gaussian field is set to $p=2$ (upper panel) and $p=8$ (lower panel). Other parameter settings are kept as: $k/\Omega /\sigma /\gamma /=1$, $T=0.5$, $g=10^{-4}$.

In Fig. 3, we disclose the influence of coupling intensity $\lambda$ of the Fock state, for the lower ($p=2$) and higher ($p=8$) order of super-Gaussian field on the dynamics of quantum correlations and entropy in the two-qubit state. In agreement with Fig. 2, the state exhibits non-zero discord, entanglement, non-classical correlations, and entropy. With time, quantum correlations in the state get induced because of the action of the Fock-state cavity and super-Gaussian field. At the final notes, all kinds of quantum correlations seem to achieve a single saturation level and are in good connection with each other. Besides this, as the entropy decreases, quantum correlations start inducing in the state, therefore, indicating an indirect relation between them. Furthermore, the coupling intensity strength not only regulates the behavioral dynamics of the system but also the initial level of quantum correlations and entropy in the system. For example, for lower $\lambda$ values, the initial quantum correlation level is enough higher (e.g. at $\lambda =1$), however, the opposite can be seen for the higher $\lambda$ values. Hence, the Fock-state cavity and super-Gaussian field are useful resources at weak coupling regimes under dephasing effects. Unlike this, the coupling strength of classical fields has been noticed to prevail non-Markovianity, however, at the final notes, quantum correlations are completely degraded^71^. Note that the differing coupling intensity strength of the Fock-state field is observed to not influence the final saturation levels of the quantum correlations and entropy function. However, the rate of generation of quantum correlations in the two-qubit state remains faster at the weak coupling regimes compared to the strong coupling regimes. Besides, as $\lambda$ increases, the generation of quantum correlations at first remains steady, however, faces a sudden transition to a higher final saturation level. In the case of the order of the super-Gaussian field, one can easily deduce that parameter *p* highly regulates the time of achieving final saturation values. For example, for the low values $p=2$, the quantum correlations functions achieve their final saturation levels by taking a longer time compared to the higher order of the Super-Gaussian field $p=8$, therefore, also agrees with the results obtained in Fig. 2. Likewise, this can be deduced for the LE function, as for $p=8$, the suppression of the entropy remains higher, while for $p=2$, entropy decrease takes a longer time. Finally, it is worth mentioning that in the current conditions, a maximal quantum correlated state along with zero disorder has been yielded.

### The impact of Ising coupling interaction of the state

**Figure 4:**
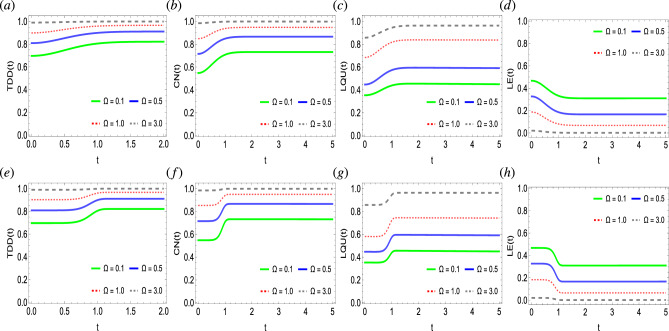
Dynamics of trace-distance discord (**a**,**e**), concurrence (**b**,**f**), local quantum uncertainty (**c**,**g**), and linear entropy (**d**,**h**) as functions of Ising coupling strengths $\Omega$ against time in a system of two qubits simultaneously influenced by Fock-state cavity, classical dephasing and super-Gaussian field when the order of the super-Gaussian field is set to $p=2$ (upper panel) and $p=8$ (lower panel). Other parameter settings are kept as: $k/T/\lambda /\gamma /\sigma /=1$, $g=10^{-4}$.

We investigate the influence of Ising coupling strengths $\Omega$ on the dynamics and generation of quantum correlations in a two-qubit state when exposed to a Fock-state cavity, super-Gaussian field, and two independent local environments (Fig. 4). We specifically disclose the impact of the lower and higher order of the super-Gaussian field ($p=2,8$ respectively) when jointly employed with the different fixed values of Ising coupling strengths. Initially, it can be readily seen that the two-qubit state remains more quantum correlated as compared to that seen in Figs. 2, and 3, hence, suggesting it is more appropriate for quantum information processing protocols. Unlike the previous cases, for $\Omega =3$, the state becomes nearly maximally entangled according to the TDD and CN functions. In comparison, the LQU measure remains weaker and shows that even for $\Omega =3$, the initial maximal non-classical correlations bound does not reach and does not agree with TDD and CN measures. On the contrary, for any high values of photon state (Fig. 2) and coupling strength (Fig. 3), the state did not achieve the maximal quantum correlations bound. It is crucial to notice that the role of the Ising interaction is opposite to that found for the photon state and coupling strength parameters. As seen for the higher photon state and coupling strength, the associated initial levels of quantum correlations appeared later and remained inversely correlated. In contrast, for the increased Ising strength, the initial, as well as the latter degree of quantum correlations in the state, become enhanced faster, hence, suggesting a direct correlation between them, and in particular, for $\Omega =3$, the state becomes maximally quantum correlated. In the case of the LE measure, close results are demonstrated, i.e., for higher $\Omega$ values, the entropy decreases, and vice versa. Note that for $\Omega =3$, entropy in the system completely vanishes, therefore, allowing the quantum correlations to reach their maximal bounds. Furthermore, the order of the Gaussian noise has been witnessed to influence the behavioral dynamics of quantum correlations and entropy. For example, for the low-order super-Gaussian field, the quantum correlation functions achieve their asymptotic values monotonically. However, for the higher order of the fields, after a steady state constant flow, a sudden transition to the final saturation levels has been witnessed. Besides, for the case of the lower order of the super-Gaussian field, the slopes of TDD, CN, LQU, and LE functions take a longer time to achieve the final saturation levels while the opposite can be seen for the higher order of the field. Therefore, for the faster generation of quantum correlations higher order of the super-Gaussian field is efficient.

### The impact of thermal interaction

**Figure 5:**
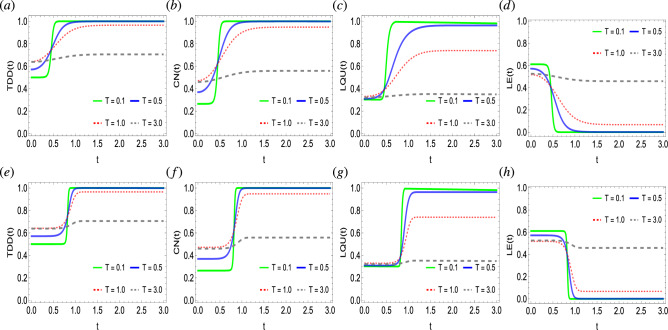
Dynamics of trace-distance discord (**a**,**e**), concurrence (**b**,**f**), local quantum uncertainty (**c**,**g**), and linear entropy (**d**,**h**) as functions of temperature *T* against time in a system of two qubits simultaneously influenced by Fock-state cavity, classical dephasing, and super-Gaussian field when the order of the super-Gaussian field is set to $p=2$ (upper panel) and $p=8$ (lower panel). Other parameter settings are kept as: $k/\Omega /\lambda /\gamma /\sigma =1$, $g=10^{-4}$.

The time dynamics of quantum correlations functions (TDD, CN, LQU,) and entropy function (LE) in a system of two qubits when coupled to joint Fock-state, super-Gaussian, and local fields is evaluated in Fig. 5. An additional emphasis on the lower and higher order of the super-Gaussian field at $p=2, 8$ and the impact of different fixed values of temperature *T* is made on the generation and behavioral dynamics of quantum correlations and entropy. Initially, the quantum correlations functions have non-zero values suggesting that the state is not separable, however, has non-zero entropy i.e., exhibits mixedness and disorder. With time, the quantum correlation functions rise monotonically and achieve a final saturation level. On the contrary, the entropy function decreases with time and finally achieves a lower saturation value. The saturation levels of the TDD, CN, and LQU functions vary, compared to the cases seen in Figs. 2 and 3. As seen for the higher temperature values, the slopes of the quantum correlations seem suppressed. Hence, suggesting that high *T* values strongly resist higher quantum correlation generation in the state. For example, when compared to the slopes of ${T}=0.1, 0.5$, where the state reached maximal quantum correlations limit, the slopes for ${T}=1, 3$ show lower generated quantum correlations in the state. This contradicts the results obtained in Figs. 2 and 3 for the photon state $\gamma$ and coupling strength $\lambda$ respectively, where for the different values of each parameter, quantum correlations function converges at a single point. Besides, the variation in the degree of quantum correlations preserved by the system at the final notes remains insignificant for temperatures lower than 1. However, for ${T}>1$, the variation in the degree of quantum correlations becomes too large, for example, see the final preserved levels by the state for ${T}=1, 3$. Besides, the entropy is enhanced by the increasing values of the temperature of the coupled fields and vice versa. Likewise, the variation in the entropy remains the least for ${T}<1$ and the opposite occurs for ${T}>1$. Besides, for the decreasing entropy values, the quantum correlation functions increase with time, hence, suggesting an inverse correlation between them and agreeing with the results of Figs. 2, 3 and 4. The fact that the higher order of the super-Gaussian field causes a sudden transition of quantum correlation slopes to higher values remains consistent with the previously obtained results in Figs. 2, 3 and 4.

### The impact of the pulse rise time of the super-Gaussian beam

**Figure 6:**
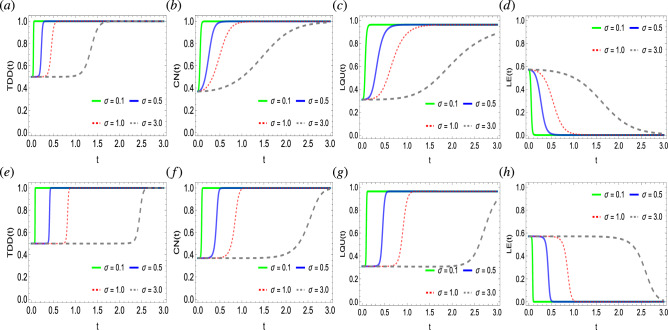
Dynamics of trace-distance discord (**a**,**e**), concurrence (**b**,**f**), local quantum uncertainty (**c**,**g**), and linear entropy (**d**,**h**) as functions of pulse rise time $\sigma$ against time in a system of two qubits simultaneously influenced by Fock-state cavity, classical dephasing and super-Gaussian field when the order of the super-Gaussian field is set to $p=2$ (upper panel) and $p=8$ (lower panel). Other parameter settings are kept as: $k/\Omega /T/\gamma /\sigma =1$, $\lambda =2$, $g=10^{-4}$.

In Fig. 6, we discuss the influence of pulse-rise time $\sigma$ of the super-Gaussian field on the dynamics of quantum correlations and entropy in a system of two qubits when exposed to Fock-state cavity and local environments. Specifically, the influence of the pulse-rise time of the super-Gaussian beams on TDD, CN, LQU, and LE functions when assumed in the lower and higher order regimes, i.e., $p=2, 8$, respectively. Initially, each function TDD, CN, and LQU has a similar initial level of quantum correlations which contradicts the previous cases from Figs. 2, 3, 4 and 5. Hence, the varying pulse-rise time $\sigma$ does not influence the initial level of quantum correlations in the state. However, the latter dynamical maps of quantum correlations function greatly become varied for the different pulse-rise times of the super-Gaussian field. The TDD function remains strengthened under the decoherence effects caused by the Fock-state cavity and super-Gaussian field. Besides, a similar amount of entropy for different values of $\sigma$ has been witnessed and, therefore, remains in close connection with the quantum correlations functions. The later dynamics of the entropy become changed for the varying pulse-rise time of the super-Gaussian field. Besides, the pulse-rise time of the field seems to negatively affect the generation of quantum correlations in the state. As can be seen for lower $\sigma$ values, quantum correlation functions achieve their final saturation levels faster when compared to the higher pulse-rise time values of the super-Gaussian field. Besides, for the higher order of the super-Gaussian field, for initial interaction time, quantum correlation generation does not take place. However, after a specific interval of time, the generation of quantum correlation for higher orders suddenly shifts towards the higher values. In contrast, for the lower order values, the quantum correlations generation takes place steadily in the state. In the case of entropy, for higher orders, the entropy suddenly becomes reduced to a specific lower value and so the opposite occurs for the lower order of the field. It is noticeable that the final saturation values remain independent of the pulse-rise time and order of the super-Gaussian fields. Besides, the state in the current case does not achieve the final maximal bound of quantum correlations.

### The impact of dipole–dipole interaction of the state

**Figure 7:**
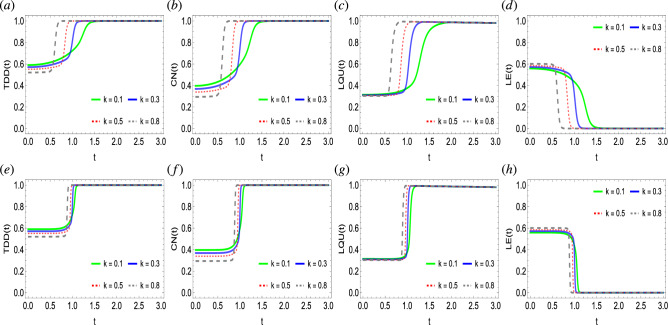
Dynamics of trace-distance discord (**a**,**e**), concurrence (**b**,**f**), local quantum uncertainty (**c**,**g**), and linear entropy (**d**,**h**) as functions of dipole–dipole interaction strength *k* against time in a system of two qubits simultaneously influenced by Fock-state cavity, classical dephasing, and super-Gaussian field when the order of the super-Gaussian field is set to $p=2$ (upper panel) and $p=8$ (lower panel). Other parameter settings are kept as: $\Omega /T/\gamma /\sigma =1$, $\lambda =2$, $g=10^{-4}$.

Figure 7 analyses the dynamics and generation of quantum correlations and suppression of the entropy in the two qubits when coupled with the Fock-state cavity, super-Gaussian beams, and local fields. The behavioral dynamical maps of TDD, CN, LQU, and LE functions are examined for different fixed values of dipole–dipole interaction strength *k* when the lower and higher orders of the super-Gaussian field ($p=2, 8$) are considered. Initially, the state seems partially quantum correlated and exhibits entropy disorder according to the quantum correlations and entropy functions. With time, quantum correlations in the state are created and entropy suppression occurs. The speed of generation of quantum correlations and rate of suppression of entropy depends upon the dipole–dipole interaction strength. For the higher dipole-dipole interaction strengths ($k=0.8$), the state quickly achieves the maximal bound of quantum correlations. For the lower dipole–dipole coupling strength ($k<2$), maximal quantum correlations in the state get generated, however, it takes a longer time. In contrast, for the higher *k* values, entropy disorder becomes completely zero faster, and so the opposite is observed for lower *k* values. Hence, the dipole–dipole interaction strength is directly correlated with quantum correlation generation but has an inverse correlation with entropy disorder in the system. Besides, for the higher super-Gaussian order, quantum correlations and entropy evolve steadily, however, at a critical point in time, the slopes of quantum correlations face a sudden transition towards the maximal quantum correlations saturation levels. Likewise can be interpreted for the entropy function too. It is noticeable that for different fixed values of the dipole–dipole interaction strengths, the initial levels of the quantum correlations can be controlled. As can be seen for the different values of dipole–dipole interaction strength, the initial values of the quantum correlations and entropy functions, show variations.

### Different limits of the classical dephasing strengths

**Figure 8:**
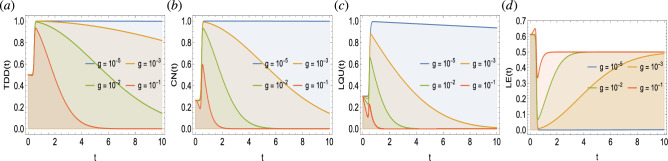
Dynamics of trace-distance discord (**a**), concurrence (**b**), local quantum uncertainty (**c**), and linear entropy (**d**) as functions of OU noise parameter *g* against time in a system of two qubits simultaneously influenced by Fock-state cavity, classical dephasing, and super-Gaussian field. Other parameter settings are kept as: $\Omega /T/\gamma /\sigma /k=1$, $\lambda =2$.

In Fig. 8, we primarily discuss the impact of classical dephasing on the dynamics of biqubit quantum correlations and entropy. In particular, we are interested in presenting certain limits of the classical dephasing which could be used to avoid the decoherence effects and preserve quantum correlations. In this regard, the TDD, CN, and LQU functions remain non-maximal at the onset but become high after some specific time. It is noticeable that the preservation perspective of the named functions further depends upon the strength of the classical dephasing. For example, for $g=10^{-5}$, the TDD and CN function becomes maximal and preserves the maximization for the later interval of time. This remains true for the LQU function except for the fact that it does not reach the maximal bound. For the stronger dephasing of the classical field, the non-classical correlations become maximal/high but face a gradual decrease and seem to vanish completely, for example, see the slopes for $g=\{10^{-3},10^{-2},10^{-1}\}$. The entropy function LN remains in good agreement with the TDD and CN function and shows that for the lower dephasing, the entropy disorder vanishes and vice versa. In comparison, the TDD function remains more preserved compared to the CN and LQU. The LQU remains more susceptible to external classical dephasing and is easily lost. It is quite interesting that even in the presence of an external classical dephasing field, the current physical model under various decoherence strengths, can generate non-classical correlations and suppress entropy disorder.

### Deviation between the inclusive measures

From the above discussion, it is clear that the inclusive measures are not equivalent in terms of quantifying the degree of quantum correlations in the state. To find the degree of deviation between them, we implement some analytic relations. Besides, the following analytical work will also analyze the deviation of the state from a maximally entangled state. We define some parameters as tools to evaluate the fractional deviation of TDD, CN, LQU, and LE from each other. Let the parameter is defined as: $J_{ab}=(1-ab)$ where $ab \in \{TDD, CN, LQU, LE\}$, respectively. Moreover, the named equation if results into 0 will represent the maximal entanglement while 1 will represent the complete disentanglement state of the system. Note that the named criteria run opposite for the case of $J_{LE}$. For the inter-comparison of the measures used in this study, we get the equations

24 $$\begin{aligned} \Delta J_{TDD-ij}=&|J_{TDD}-J_{ij}|,&\text {when}&\,{ij}=\{ CN, LQU, LE\}, \end{aligned}$$

25 $$\begin{aligned} \Delta J_{CN-kl}=&|J_{CN}-J_{kl}|,&\text {when}&\,{kl}=\{TDD, LQU, LE\}, \end{aligned}$$

26 $$\begin{aligned} \Delta J_{LQU-mn}=&|J_{LQU}-J_{mn}|,&\text {when}&\,{mn}=\{TDD, CN, LE\}, \end{aligned}$$

27 $$\begin{aligned} \Delta J_{LE-pq}=&|J_{LE}-J_{pq}|,&\text {when}&\,{pq}=\{TDD,CN, LQU\}. \end{aligned}$$

The above equations will evaluate how much these functions deviate from each other at a specific interval of time. For example, for $t=0.1$, we have derived Table 1, showing the relative deviations in terms of percentage.

**Table 1:** The inter-deviation of TDD, CN, LQU, and LE when $\gamma =1$, $T,/t=0.1$, $g=1$, $k=1$, $J=1$, $\sigma =1$, $g=2$, and $p=2$.

TDD	CN	LQU	LE	$J_{TDD}$	$J_{CN}$	$J_{LQU}$	$J_{LE}$	P$\%$
0.49	0.256			0.509	0.75			$\Delta _{TDD-CN}$ = 23.56%
0.49		0.24		0.509		0.76		$\Delta _{TDD-LQU}$ = 25.23%
0.49			0.62	0.509			0.38	$\Delta _{TDD-LE}$ = 12.52%
0.49	0.256			0.509	0.75			$\Delta _{CN-TDD}$ = 25.46%
	0.256	0.24			0.75	0.76		$\Delta _{CN-LQU}$ = 1.66%
	0.256		0.62		0.75		0.38	$\Delta _{CN-LE}$ = 36.09%
0.49		0.24		0.509		0.76		$\Delta _{LQU-TDD}$ = 25.23%
	0.256	0.24			0.75	0.76		$\Delta _{LQU-CN}$ = 1.66%
		0.24	0.62			0.76	0.38	$\Delta _{LQU-LE}$ = 37.76%
0.49			0.62	0.509			0.38	$\Delta _{LE-TDD}$ = 12.52%
	0.256		0.62		0.75		0.38	$\Delta _{LE-CN}$ = 36.09%
		0.24	0.62			0.76	0.38	$\Delta _{LE-LQU}$ = 37.76%

Note that P% presents the percentage of the deviation between the respective measures.

Table 1 shows that the lowest deviation occurs between the CN and LQU while the greatest deviation occurs between the LQU and LE. Besides, the $J_{LQU}$ function under the given criteria shows that the state is more entangled at $t=0.1$. Note that the functions $J_{ab}$ with $ab \in \{TDD, CN, LQU, LE \}$ demonstrate the distance between the maximal entanglement and the current state.

**Figure 9:**
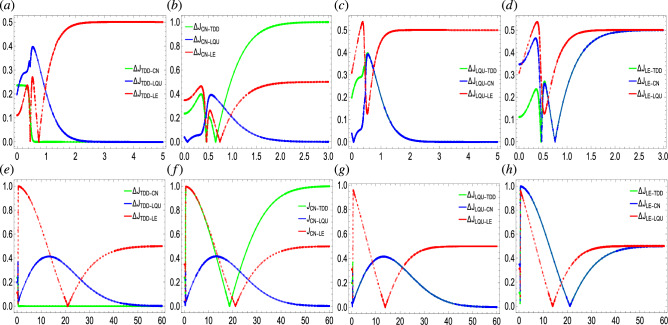
The inter-deviation dynamics of TDD, CN, LQU, and LE when $T/t=0.1$, $g/k/\Omega /\sigma =1$, $g/p=2$, $\gamma =1$ (top panel) and $\gamma =10^{-3}$ (bottom panel).

In Fig. 9, the deviation dynamics between the functions TDD, CN, LQU, and LE are plotted with time under weak ($g=10^{-3}$) and strong dephasing $(g=1)$. First, the deviation maps under the weak and strong dephasing seem completely different than each other. For example, under strong dephasing, the saturation of the graphs at a stable value occurs quickly in comparison. Various types of behaviors such as increasing, decreasing, and stable dynamics of the slopes are recorded with time, depending upon the functions involved. From the current results, it can also be deduced that the degree of deviation of the functions is also dependent upon the degree of dephasing. For example, in Fig. 9a,e, for the strong dephasing, the slopes oscillate and then attain the highest ($\Delta J_{TDD-LE}$) or the lowest values ($\Delta J_{TDD-CN}/\Delta J_{TDD-LQU}$). On the contrary, for the weak dephasing, the deviation function $\Delta J_{TDD-LE}$ at first accelerates and achieves the highest value, death, and a stable value respectively. The $\Delta J_{TDD-CN}$ shows a small variation that quickly vanishes unlike that occurs during the strong dephasing. Likewise can be explored for Fig. 9b,f where there were abrupt rise and fall under strong dephasing and then slopes achieves the stabilization while a rise, death, and constant stabilization is achieved respectively in the case of weak dephasing with time. Note that $\Delta J_{CN-TDD}$ shows the highest deviation in comparison. The deviation under strong dephasing first increase and then decrease to a zero or constant value, as seen in Fig. 9c. On the contrary, the slopes show a sudden rise ($\Delta J_{LQU-LE}$) influenced by weak dephasing (Fig. 9g) and then achieves a similarly high value as that obtained under strong dephasing. On the other hand, $\Delta J_{LQU-TDD}$/$\Delta J_{LQU-CN}$ both achieves zero saturation level in both weak and strong dephasing. Similar results can be seen in Fig. 9d,h however, with repeated deaths under strong dephasing. Note that in the current case, there is no zero-saturation level obtained in the case understudy, meaning that there is always deviation between the inclusive functions.

Finally, we provide the significance of the current study. Here, we demonstrated the significance of the Fock-state cavity, super-Gaussian field accompanying a classical dephasing field to control and generate quantum correlations in a two-qubit system. Several varying results are obtained showing the partial and maximal quantum correlations generation along with the entropy suppression. For this sake, various types of physical models have been proposed previously^72–75^. However, we find the states either undergoing losses or resulting in partial non-classical correlations generation. We present a model where a two-qubit state exhibits lower levels of initial quantum correlations, however, with time, quantum correlations get generated in the state and do not face any losses. Therefore, the Fock-state cavity when interfered with the super-Gaussian field becomes a vital resource for quantum correlations generation even under the presence of classical dephasing. Under the effects of solely employed Fock-state cavity field, the two-qubit system remains completely separable for a specific interval of time^37^. Interestingly, with the help of the super-Gaussian field, the initial duration of the separability of the state vanished in the current case. In addition, the current model does not support non-Markovianity and the generation of quantum correlations takes place monotonically, either with a higher or lower rate depending upon the stimulation of the order of the super-Gaussian field. Finally, in any case, the state never gets separable for any values of the parameters of the current configuration.

## Conclusion and outlook

We addressed the role of jointly deployed Fock state cavity, super-Gaussian beam, and local dephasing on the dynamics and generation of quantum correlations and suppression of the entropy in a two-qubit state. Specifically, we probed quantum discord, entanglement, and non-classical correlations using TDD, CN, and LQU functions. To investigate mixedness disorder, we employed the LE function. The employed functions are characterized by different parameters of the current physical model and the resultant generation of quantum correlations and entropy suppression is discussed in detail. The joint impact of Fock-state and super-Gaussian fields when influenced by the decoherence effects on the dynamics of the two-qubit system is also closely studied.

We show that the joint configuration of the Fock-state cavity and super-Gaussian field remains a vital resource for the generation of quantum correlations and suppression of the mixedness disorder in a two-qubit system when influenced by classical dephasing. We find that when the quantum correlations functions are characterized by lower values of the photon states of the Fock-state cavity; the state approaches a maximal bound of quantum correlations faster. The higher coupling strength of the Fock-state cavity along with the super-Gaussian field negatively affects the quantum correlations generation, however, only for a specific interval of time. On the contrary, the Ising interaction and dipole–dipole interaction strengths of the Fock-state cavity have been found to greatly enhance quantum correlations and suppress the mixedness disorder in the system. The temperature of the coupled configuration remained a vital factor and for $T<1$, maximal non-classical correlations bound is readily achieved while entropy completely vanishes. The pulse-rise time of the super-Gaussian field also resists the initial generation of quantum correlations and sustains entropy in the state for a short duration of time. Most importantly, the order of the super-Gaussian field has been found initially sustaining quantum correlations at a steady rate but causing a sudden transition of the quantum correlations to a higher level.

In comparison, the generation of quantum correlations has been found in trade-off relation with the entropy. As seen when entropy gets decreased, quantum correlations become induced in the state. In comparison, quantum discord has been found the most strengthened among the others followed by entanglement. The non-classical correlation estimated using the LQU function remains fragile and is generated to lower values and at a slower speed. Finally, under certain limits of the classical dephasing, the decreased temperature, higher order super-Gaussian field, Ising, and dipole–dipole interaction strengths can play a vital role in inducing entropy-free and maximally quantum correlated two-qubit state.

In the future, we expect our presented model to be resourceful for the deployment of various quantum protocols. For example, the current model can be utilized to reduce the fidelity and errors, thus, an improved quantum state transfer can be obtained compared to using microwave cavity memories^76^. We believe an improved universal algorithms performance can be obtained utilizing the single-mode cavity assisted by various interactions studied here, compared to the simple ones presented in Ref.^77^. Besides, the practical empowerment of super-Gaussian beams instead of the simple Gaussian ones can yield enhanced photon statistics, coherence, and certain correlation functions^78^. Using the concept of circuit knitting such as entanglement forging, circuit cutting, and quantum embedding methods can be applied to the current configuration to design minor non-classical circuits on a quantum computer with improved quantum information preservation limits^79^. In particular, quantum correlations detected beyond entanglement in the current model will provide a high level of security in the quantum key distribution protocol.

## Data Availability

The authors confirm that the data supporting the findings of this study are available within the article.
