# Author Correction: Extremal quantum correlation generation using a hybrid channel

**DOI:** 10.1038/s41598-024-52466-w

**Published:** 2024-01-23

**Authors:** Atta ur Rahman, Hazrat Ali, S. M. Zangi, Cong‑Feng Qiao

**Affiliations:** 1 School of Physics, University of Chinese Academy of Science, Yuquan Road 19A, Beijing, 100049 China; 2 Abbottabad University of Science and Technology, Havellian, KP 22500 Pakistan; 3 School of Physics and Astronomy, Yunnan University, Kunming, 650500 China

Correction to: *Scientific Reports* 10.1038/s41598-023-43811-6, published online 03 October 2023

The original version of this Article contained errors.

Equation 12 was incorrectly given as:

$$\begin{array}{c}\eta (t)=\frac{1}{g}[g\tau -1+{\text{exp}}\,[-g\tau ]]\end{array}$$

The correct Equation 12 appears below.

$$\eta (t)=\frac{1}{g}[gt-1+{\text{exp}}\,[-gt]]$$

In addition, in the Physical model section, under the subheading ‘Classical noisy procedure’.

“Note that $$\hat{{\rho }_{32}^{*}}=\hat{{\rho }_{23}}={\rho }_{23}{\text{exp}}\,[-\frac{4(\gamma t+{e}^{\gamma (-t)}-1)}{\gamma }]$$.”

now reads:

“Note that $$\hat{{\rho }_{32}^{*}}=\hat{{\rho }_{23}}={\rho }_{23}{\text{exp}}\,[-\frac{4(gt+{e}^{g(-t)}-1)}{g}]$$.”

The original Article has been corrected.

